# Delayed traumatic intracerebral hemorrhage associated with dolichoectasia of the middle cerebral artery

**DOI:** 10.1016/j.radcr.2022.01.039

**Published:** 2022-02-04

**Authors:** Aito Watanabe, Satoshi Tsutsumi, Hiroki Sugiyama, Senshu Nonaka, Hidehiro Okura, Hisato Ishii

**Affiliations:** Department of Neurological Surgery, Juntendo University Urayasu Hospital, Japan

**Keywords:** Delayed traumatic intracerebral hemorrhage, Dolichoectasia, Middle cerebral artery, Natural history

## Abstract

A 74-year-old man tripped while walking. He had not been administered antiplatelet or anticoagulation therapy. At presentation, the patient was well-oriented, with a blood pressure of 130/91 mmHg, while present with tetraplegia and numbness in the upper extremities. Blood work revealed normal findings, while magnetic resonance imaging of the cervical spine revealed severe cord compression at the C3/4 and C4/5 levels. Cranial computed tomography (CT) showed elongated masses in the Sylvian fissures without intracranial hemorrhage. CT taken 2 days later revealed an intraparenchymal hemorrhage located mainly in the right putaminal region, while the patient showed no signs of neurological deterioration. Three-dimensional CT angiography (3D CTA) demonstrated marked ectasia and elongation in the right internal carotid, bilateral middle cerebral, and left anterior cerebral arteries. The patient was conservatively managed. Repeat 3D CTA performed 3 months later showed no de novo abnormalities in the ectatic cerebral arteries. It is assumed that the delayed traumatic intracerebral hemorrhage was caused by disruption of the perforating vessels arising from the ipsilateral dolichoectatic middle cerebral artery. Periodical surveillance neuroimaging is recommended for patients with head trauma who are simultaneously diagnosed with incidental dolichoectasia, especially when complicated with cervical cord injury.

## Introduction

Delayed traumatic intracerebral hemorrhage (DTIH) refers to the appearance of hemorrhage, usually within 24 or 48 hours of head trauma, in parenchymal regions of the brain that were normal in appearance on computed tomography (CT) performed shortly after injury [1,2]. Although it has been frequently reported since 1970, the time from trauma to hemorrhage and diagnosis has not been well defined [1], [2], [3], [4], [5], [6], [7], [8]. It is estimated to occur in 0.27% of head injuries or in 1%-8% of patients with severe head injuries [2,3].

Dolichoectasia, a distinct manifestation of arterial wall remodeling, is characterized by an increase in the diameter and length of major cerebral arteries. Diverse clinical manifestations can be caused by dolichoectasia, including ischemic stroke, intracerebral hemorrhage, subarachnoid hemorrhage, small vessel disease, cranial nerve or brainstem compression, and hydrocephalus [9]. Compared to the anterior circulation, dolichoectasia is more frequent in the vertebrobasilar system [10]. Dolichoectasia affecting the anterior and middle cerebral arteries has rarely been documented [11], [12], [13], [14]. The natural history of dolichoectasia is not well understood. They are commonly found as radiographic findings, although some show progressive worsening [15,16].

To the best of our knowledge, no study has documented the relationship between head trauma and preexisting dolichoectasia.

Herein, we present a unique case of DTIH associated with dolichoectasia of the middle cerebral artery.

## Case report

**Figure 1:**
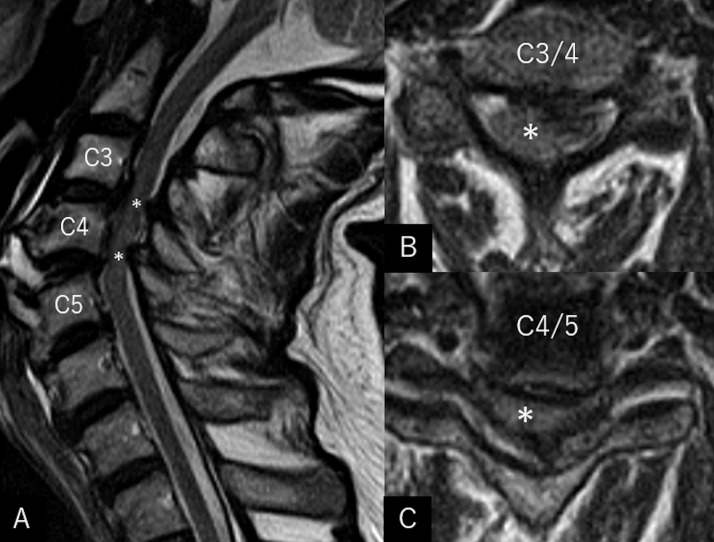
Sagittal (A) and axial (B, C) T2-weighted magnetic resonance images of the cervical spine showing severe cord compression at the C3/4 and C4/5 levels (asterisk).

**Figure 2:**
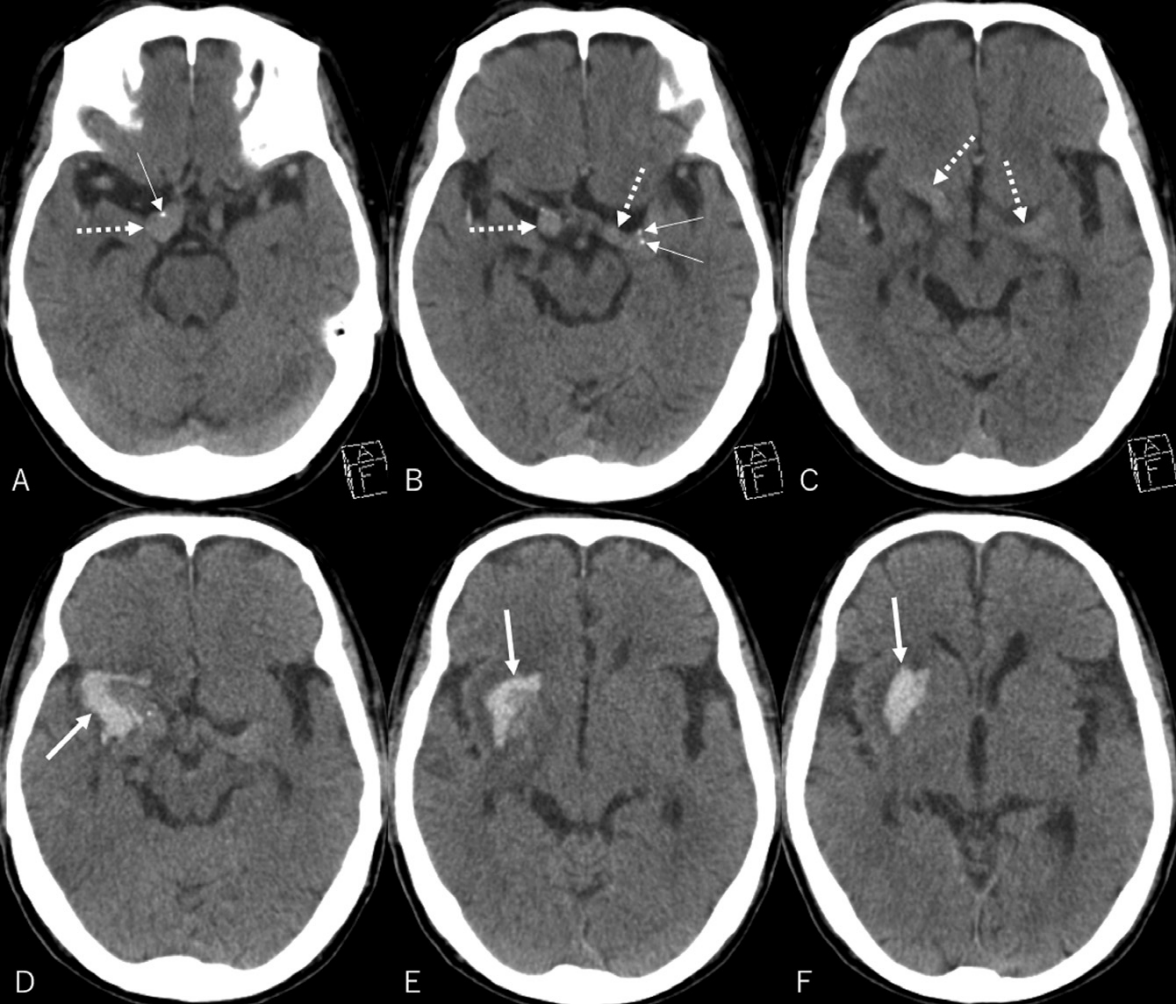
(*A-C*) Non-contrast axial computed tomography taken at presentation showing elongated masses lying in the bilateral sylvian fissures (*dotted arrows*), accompanying punctate calcifications (*thin arrows*), without hemorrhagic changes. (*D-F*) Non-contrast axial computed tomography taken 2 days later showing an irregularly shaped intraparenchymal hemorrhage, 30 × 18 × 35 mm in maximal dimension, located mainly in the right putaminal region (*arrow*).

**Figure 3:**
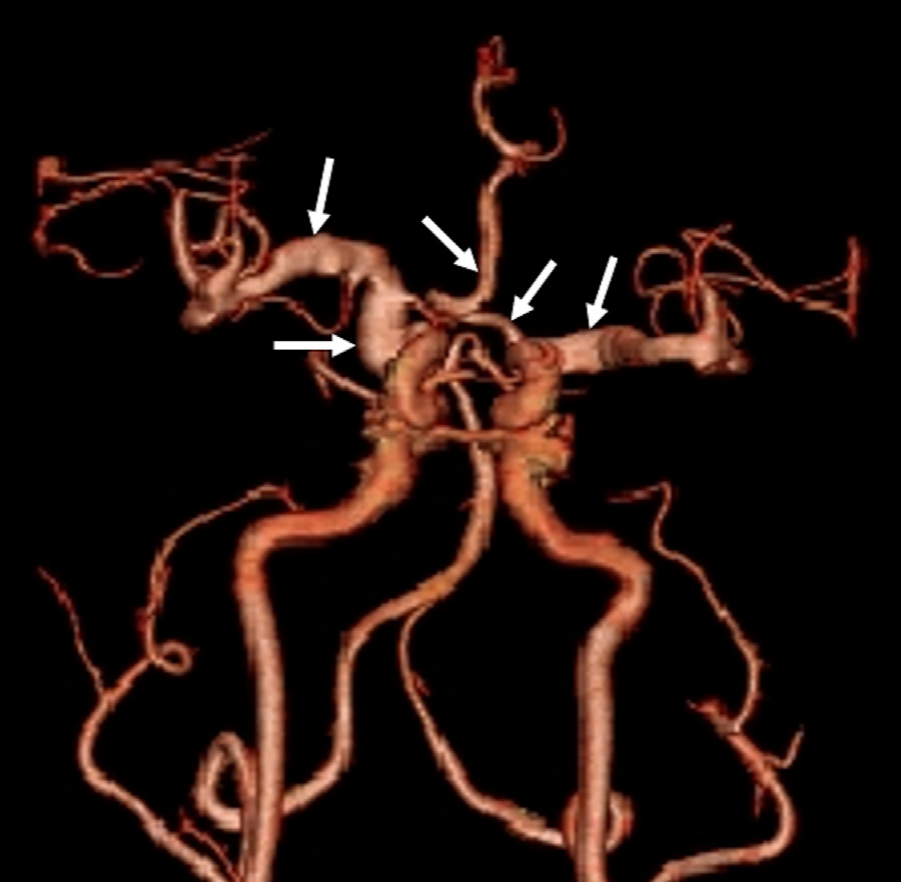
Three-dimensional computed tomography angiography, anteroposterior view, showing abnormal ectasias and elongations in the paraclinoid portion of the right internal carotid, bilateral middle cerebral, and left anterior cerebral arteries (*arrows*).

**Figure 4:**
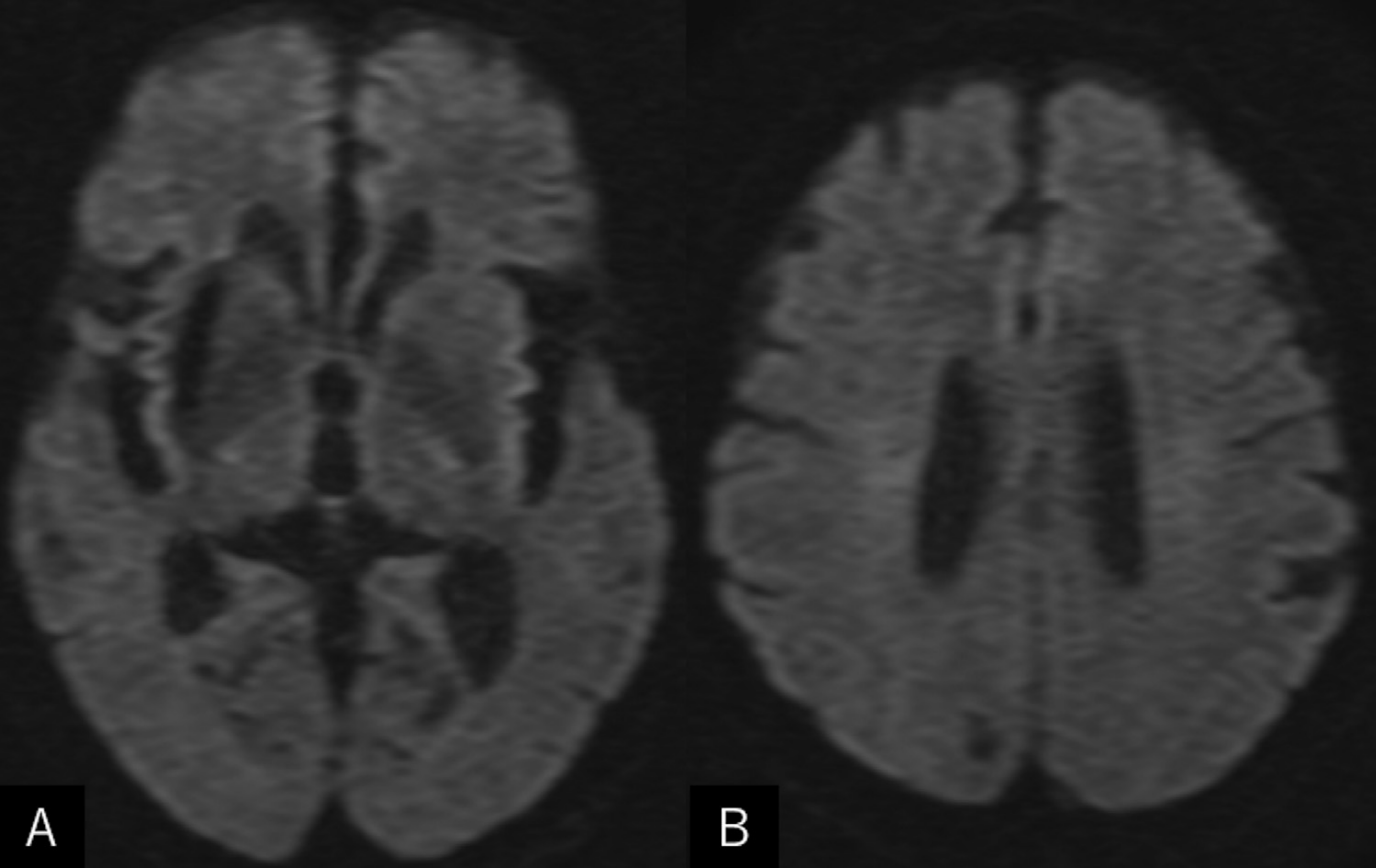
(*A, B*) Diffusion-weighted magnetic resonance images showing absence of infarcts in super-acute phase.

**Figure 5:**
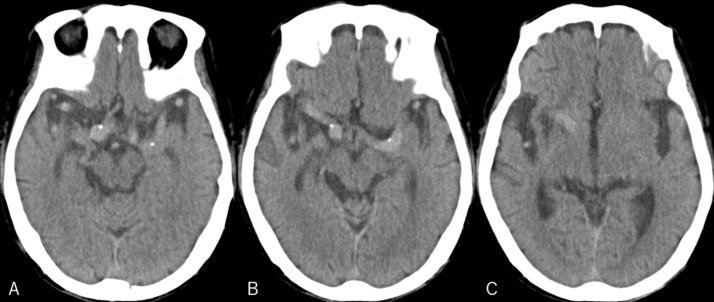
(*A-C*) Non-contrast axial computed tomography taken 3 months later showing resolution of the intracerebral hemorrhage.

**Figure 6:**
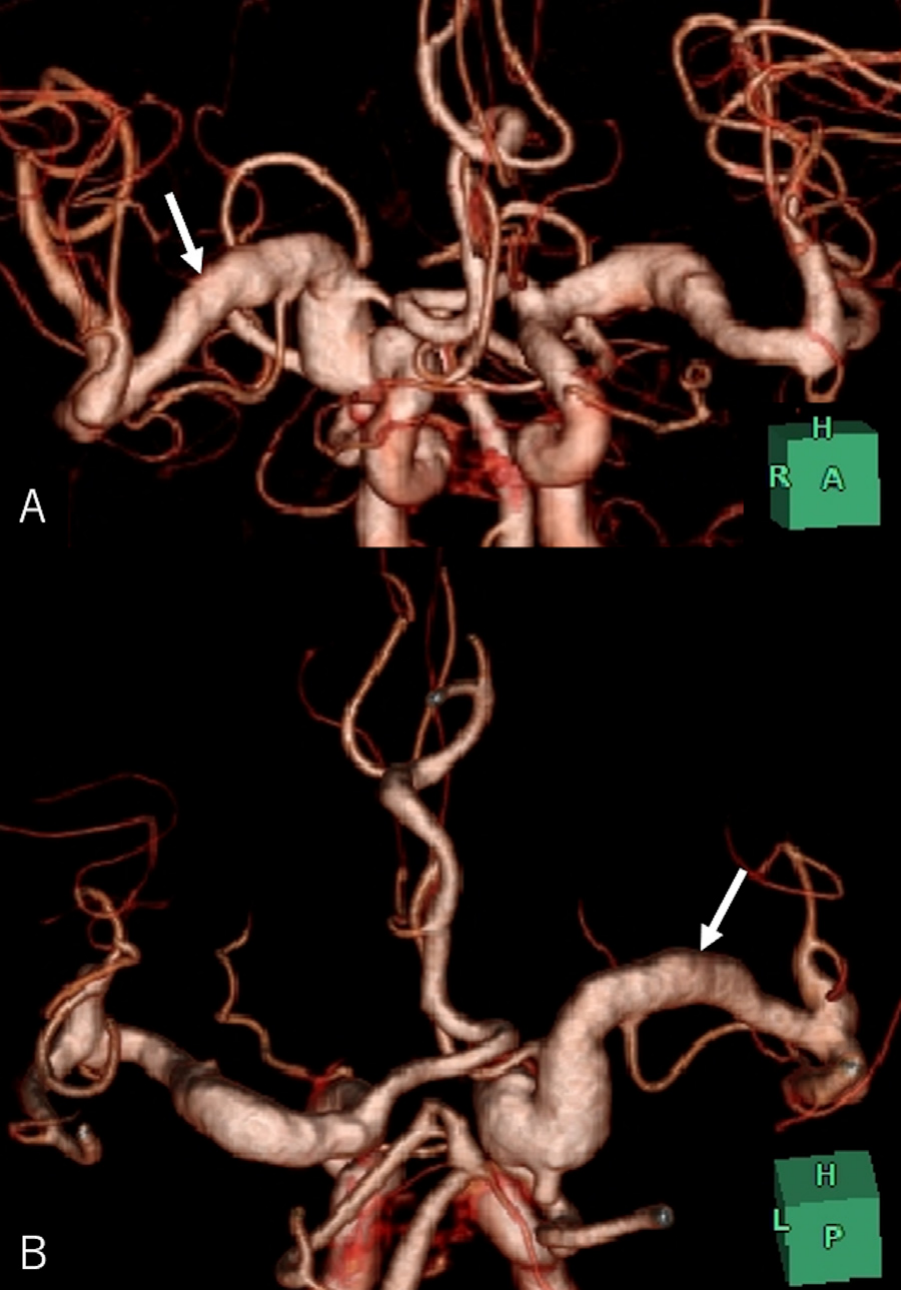
Three-dimensional computed tomography angiography, anteroposterior (*A*) and posteroanterior (*B*) views, performed 3 months after injury, showing walls of the dolichoectatic right middle cerebral artery without de novo abnormalities (*arrow*).

A 74-year-old man who tripped while walking suffered from blunt forehead trauma was transported to our institution on the same day. The patient had a history of diabetes mellitus, hypertension, dyslipidemia, and acute myocardial infarction. He had not been administered antiplatelet or anticoagulation therapy. At presentation, the patient was alert and well-oriented, with the blood pressure at 130/91 mmHg. He had bruises in the right forehead, cheek, and nose, and was present with tetraplegia and numbness in the upper extremities. Blood work revealed normal findings, while magnetic resonance imaging (MRI) of the cervical spine revealed severe cord compression at the C3/4 and C4/5 levels (Fig. 1). Cranial CT showed elongated masses in the bilateral Sylvian fissures, accompanied by punctate calcifications. No intracranial hemorrhagic changes were observed (Fig. 2A-C). However, a surveillance CT taken 2 days later revealed an irregularly shaped intraparenchymal hemorrhage, 30 × 18 × 35 mm in maximal dimension, located mainly in the right putaminal region (Fig. 2D-F). Since hospitalization, the patient had stable blood pressure and displayed no signs of neurological deterioration. Three-dimensional CT angiography (3D CTA) demonstrated abnormal ectasia and elongation in the paraclinoid portion of the right internal carotid, bilateral middle cerebral arteries, and left anterior cerebral arteries (Fig. 3). In addition, no infarcts in the super-acute phase were identified on cerebral MRI (Fig. 4). The patient was conservatively managed by craniocervical fixation using a Hollo vest. CT performed 3 months later revealed resolution of the intracranial hemorrhage (Fig. 5). At that time, 3D CTA did not show de novo irregularities or local protrusions in the walls of the right middle cerebral artery and those of other cerebral arteries presenting with abnormal ectasias (Fig. 6). The patient was eventually transferred to a chronic care facility, with activities of daily living confined to a wheelchair.

## Discussion

In the present case, DTIH was detected on surveillance CT performed 2 days after the injury. Despite its sizable dimension and main location in the putaminal region, the patients did not show neurological deterioration, which was probably due to the extension of the DTIH that had minimal effect on the pyramidal tract coursing through the internal capsule, in addition to masked symptoms caused by the DTIH, which was due to the spinal cord injury at the C3/4 and C4/5 levels. The time from trauma to hemorrhage and diagnosis is not well defined and may vary [1,2]. In rare instances, DTIHs may develop following asymptomatic intervals of more than 3 months [17]. Therefore, periodic surveillance neuroimaging of the brain may be recommended for patients with head trauma, especially when complicated by cervical cord injury.

In our patient, on neuroimaging, the DTIH was confined within the cerebral parenchyma without egress into the cerebral cisterns or ventricles. Furthermore, no de novo abnormalities were found in the walls of dolichoectatic arteries. This means that the DTIH might have been caused by traumatic disruption of the perforating vessels arising from the dolichoectatic middle cerebral artery, not affecting the walls of the dolichoectatic vessels during the follow-up period. DTIH is distinct from delayed intraventricular hemorrhage, a peculiar type of intraventricular hemorrhage that is thought to develop in association with moderate-to-severe traumatic brain injury [18]. In our case, such hemorrhage was not observed on repeat examinations.

In conclusion, disruption of perforating vessels arising from the dolichoectatic middle cerebral artery was assumed to cause DTIH. Periodical surveillance neuroimaging of the brain is recommended for patients with head injury, who are simultaneously diagnosed with incidental dolichoectasia, especially when complicated with cervical cord injury.

## Author Contributions

All the authors contributed equally to the study.

## Ethical Standards and Patient Consent

We declare that the present study has been approved by the institution's guidelines for human research and performed in accordance with the ethical standards laid down in the 1964 Declaration of Helsinki and its later amendments. We declare that the patient described in this study gave informed consent prior to inclusion in this study.
